# Using Pitch Feature Matching to Design a Music Tutoring System Based on Deep Learning

**DOI:** 10.1155/2022/4520953

**Published:** 2022-09-06

**Authors:** Jing Jiang

**Affiliations:** College of Music and Dance, Zhengzhou Normal University, Zhengzhou 450044, China

## Abstract

It is a challenge for the current music teaching system to carry out teaching according to the difference of score difficulty and realize automatic grading. Therefore, identifying the difficulty of music score according to pitch is the key to individualize music teaching resources. This paper summarizes and analyzes the problem of pitch feature extraction in music teaching. In the pitch extraction, the audio signal is divided into frames, and the feature matching of high-pitched content in music teaching resources is realized by smoothing the pitch sequence. In addition, the pitch feature extraction algorithm in MIDI music score files is proposed, and the pitch feature matching model is constructed. Finally, a music tutoring system based on pitch feature matching is designed, including a music score learning tool, overall structure of system, and interaction between teachers and students. Tutoring strategies include three main functions: learning suggestions of knowledge points, skills in practice and training, and learning path adjustment. This study is helpful to further improve the music teaching model and realize intelligent and personalized music learning.

## 1. Introduction

With the rise of Internet teaching, the limitations of traditional teaching methods in time and space have been solved, and virtual teaching scenes and resources have been built by the network-based teaching platform, which makes the teaching behavior no longer confined to classrooms and classrooms. An intelligent learning guidance system uses artificial intelligence technology to simulate and learn the teaching methods of human teachers and furnishes them with customized learning ways and learning ideas as per the requirements of various students. In the educational experience, we can find out about their learning propensities and inclinations through language correspondence, conduct investigation, recreation, and so on and dynamically guide learners to master knowledge [1, 2]. In recent years, artificial intelligence (AI) technology has greatly promoted the development of intelligent tutoring system, formed a standardized structural framework, and produced many excellent products in computer foundation, language, medicine, mathematics, and other disciplines [3–5].

Musical Solfeggio is a course based on mastering the basic principles of music, which aims at cultivating students' abilities of reading music, singing music, memorizing music scores, recognizing music scores, and perceiving, analyzing, expressing, and imagining music. With the continuous development of the music education system, the teaching concepts of sight, singing, practice, and listening are constantly innovated. However, teaching and training in the classroom has been adopted for many years, which is not only wasteful, yet in addition, incredibly influences understudies' advantage in learning. Albeit increasingly more sight and sound apparatuses and showing programming are utilized in the study hall of solfeggio educating, they can help instructing, while the current internet-based courses and Internet learning stages just gives rich learning assets and different learning ways for students, and it can not meet the demand of intelligent teaching [6]. At the same time, the evaluation of students' ability and achievement is still done by teachers or experts, and the current scoring method can only evaluate the difference between singing performance and standard template. However, in the process of music teaching, different music has different difficulty in singing, so there is a big error in the evaluation method that only considers the difference between template and performance [7]. In addition, the same problem as learning resources is that there is no uniform standard for the classification of music score difficulty level, and it depends on the subjective judgment of experts. In the evaluation of music teaching, the questions can only come from expert question banks or textbooks, while the individual inclination and expert level will influence the equilibrium of the trouble about the inquiries, which can not ensure the decency, exactness, and objectivity of the assessment [8, 9]. Therefore, it is also a challenge for the solfeggio teaching system to achieve automatic scoring according to the differences in score difficulty.

Therefore, this paper combines the characteristics of music, matches the characteristics of pitched content in music teaching resources, and designs an intelligent music tutorial system, which is helpful to further improve the music teaching model and realize intelligent and personalized music learning.

## 2. Research Basis of Pitch Feature Matching

### 2.1. Description of Pitch Features

Pitch is one of the most familiar note attributes, and intuitive understanding is the height of the note; as described in the previous chapter, the sound is composed of fundamental tone and overtone, and the part that determines the pitch of the sound is the fundamental tone. Physically, the vibration frequency, that is, Hertz, is used to describe the pitch of the sound. The pitch range recognized by human ears is about 20 to 20,000 Hz. Generally speaking, when people describe sounds, the higher the frequency, the higher the tone, which can be said to be a positive correlation [10].

According to the frequency, music can be divided into different pitches. The most commonly used method is the twelve-average law method, which divides an octave into twelve parts on average by a certain mathematical method of raising the power, which can improve the true performance of music and make the pitch reasonably distributed. In addition, the notation can be divided into numerical notation and alphabetic notation according to the different forms of notation symbols. The numeral notation uses numbers to indicate the pitch, and the short line under the numbers indicates the sound value, that is, the duration of notes; digital notation has the advantages of convenient presentation and low learning difficulty. By using the law of twelve averages to divide the pitch, an octave can be divided into twelve points, each of which is called a semitone; similarly, an octave can be divided into six whole tones by the law of twelve averages.

### 2.2. Analysis of Pitch Feature Extraction

**Figure 1:**
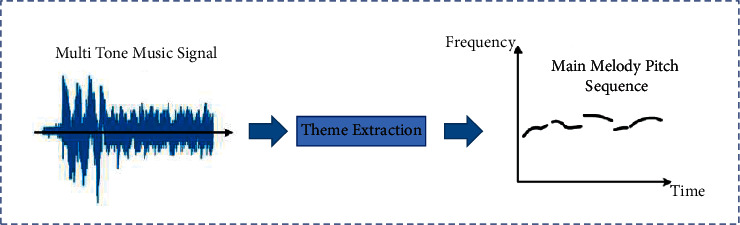
Pitch feature extraction process.

The research object of main melody extraction is polyphonic music composed of voice and one or more musical instruments. As shown in Figure 1, the target of main melody extraction is to extract the vocal pitch sequence defined as the main melody from such mixed audio signals.

**Figure 2:**
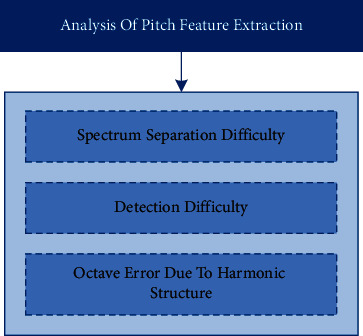
Problem analysis of pitch feature extraction.

Because the singing and accompanying note components have rich harmonic information, the harmonic characteristics of music signals can be fully utilized in the pitch estimation stage. When judging the pitch fundamental frequency, only the energy information of the fundamental frequency and its harmonic components plays a positive role, and other frequency energies are equivalent to noise here, which will interfere with the main melody extraction. However, due to the existence of harmonic characteristics, octave errors often appear in the main melody extraction results [11]. An octave error means that when the pitch is estimated, the pitch is mistakenly identified as one or more octaves different from the correct pitch, which makes it easy for people with healthy hearing to identify singing information from complex multisource music signals without being influenced by other accompaniments. However, it is extremely difficult to extract the main melody by computer, as shown in Figure 2, which is mainly caused by the following factors:

1. Multitone music signals are composed of the superposition of sound waveforms produced by all instruments in the recording. Many times these instruments are played at the same time. It is extremely difficult to separate the corresponding notes from different sound sources according to the frequency spectrum which is highly coupled and superimposed with the sound structure, and the later reverberation, echo, and other treatments will further increase the overlap of sound sources. Blurring the start and end time of the music signal makes spectrum separation more difficult.
2. Even if the fundamental frequency sequence of notes has been obtained, it is still necessary to judge which pitches belong to melody and which belong to accompaniment. When the melody is singing but there is background harmony, it is more difficult to detect.
3. Because of the existence of harmonic structure, it is easy to produce octave errors.

For the existing methods, most methods based on pitch saliency have an unavoidable problem, that is, due to the harmonic characteristics of music signals, the algorithm can easily output the fundamental frequency of an octave before and after the correct melody pitch, resulting in octave errors. While the method based on source separation is more dependent on singing energy, for strong accompaniment, it is difficult for the model to correctly separate melody and accompaniment.

Therefore, this paper combines the characteristics of music and matches the characteristics of high-pitched content in music teaching resources based on the deep learning.

## 3. Pitch Feature Matching Based on Deep Learning

### 3.1. Deep Learning Theory

**Figure 3:**
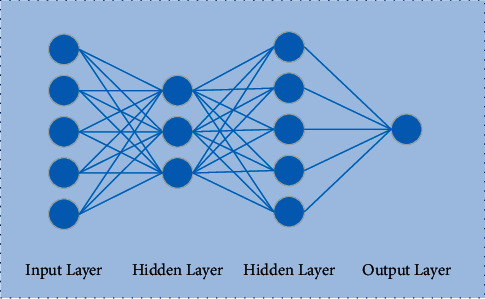
Fully connected neural network model.

The purpose of deep learning is to establish a learning model similar to a brain neural network, which is a branch of machine learning. For machine learning algorithms, there are only one or two nonlinear feature structures. However, on complex problems, such as speech signal and image processing, these shallow structures have their limitations, while deep learning builds deep nonlinear structures, which can express richer information [12, 13]. In this paper, a fully connected layer neural network is adopted, as shown in Figure 3, it is a feedforward neural network with two hidden layers.

In order to make the network learn nonlinear characteristics and increase the complexity of the model, there is usually a nonlinear activation function between each layer this paper adopts the LRelu function, as shown in formula (1), which is a variant of ReLu.

(1) $$\begin{array}{c} \text{LRelu}\left(x\right)=\left\{\begin{array}{cc} x, & x\geq 0,\\ 0.01x, & x<0. \end{array}\right. \end{array}$$

The main melody extraction in this paper is actually the task of classifying pitches. In a neural network, for binary logistic regression, the output layer can be activated by sigmoid, and the real number field can be mapped to 0 ~ 1 Range, this is the output probability of normal class. For multiclassification problems, the output layer can be activated by softmax, and its formula is as follows:

(2) $$\begin{array}{c} S_{i}=\frac{e^{z_{i}}}{\sum_{n=1}^{N}e^{z_{n}}}. \end{array}$$

The training process of the neural network is a process of gradient iterative updating. Usually, the gradient descent method is used to find the optimal solution of the objective function, which makes the loss function smaller and smaller and makes the model learn the information of deep features from a large amount of data.

### 3.2. Feature Extraction Algorithm

**Figure 4:**
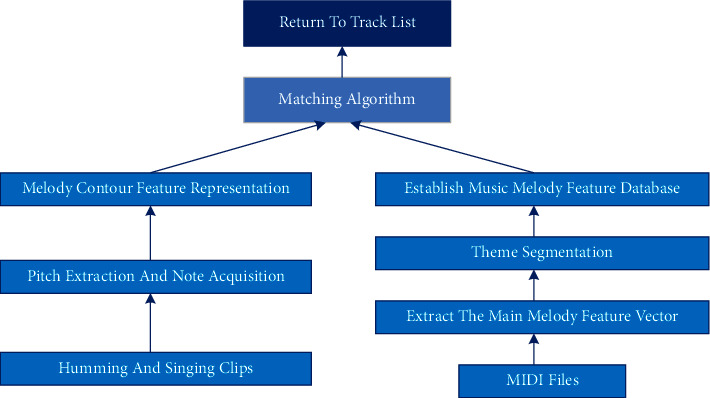
Steps of music feature extraction.

In this study, the evaluation of music is based on the MIDI music score file, while MIDI uses pitch value and duration to describe notes in a music score, so it is necessary to extract pitch features and duration of solfeggio audio. The feature vector of music is usually obtained by the main melody, and MIDI files usually include multi-track accompaniment. It is very important to extract the main melody that can represent complete music information from a multitrack MIDI melody, whose extraction steps are shown in Figure 4.

#### 3.2.1. Build Feature Vectors

Each note in the main melody corresponds to a characteristic point, which is described as follows:

(3) $$\begin{array}{c} v=\langle \text{Pitch,Time}\rangle , \end{array}$$

where Pitch is the value of pitch, and the note value is derived from 0 ~ 127; Time is the improvement of MIDI time, which represents the information of duration. The feature quantity corresponding to the main melody note sequence can be expressed as follows:

(4) $$\begin{array}{c} V=\left\{v_{1},v_{2},\cdots ,v_{n}\right\}. \end{array}$$

Among them, *V* is the sequence of note characteristic points of the whole music, *n* is the total number of notes.

Considering that there are phrases in music, the above vector can be further expressed as follows:

(5) $$\begin{array}{c} V=\left\{P_{1},P_{2},\cdots ,P_{k}\right\}. \end{array}$$

Among them, *V* is the sequence of note characteristic points of the whole music, *k* is the total number of phrases.

(6) $$\begin{array}{c} P_{i}=\left\{v_{i1},v_{i2},\cdots ,v_{\text{in}}\right\}. \end{array}$$

This feature vector can well express the melody and rhythm of the music.

#### 3.2.2. Extraction of Pitch Pitch

The notes in each MIDI track are determined by two MIDI events: note on and note off. MIDI message: XX NN KK.XX represents the status byte, which determines 8 MIDI commands and 16 MIDI channels. The commonly used MIDI command 9x (X represents channel number) indicates that the note is on, and the data byte NN immediately following represents the pitch symbol (pitch), with a value of 1–127, and two consecutive notes are opened; the second note can be omitted to open the command 8x means off. KK means the key and release force (vel) value is 0∼127. Command 9x followed by the key strength KK is 0, equivalent to note off. The Polyphony of music determines the simultaneous pronunciation of notes. In this paper, the value of the highest pitch note is selected according to the skyline algorithm, and the values of the remaining simultaneous notes are deleted.

According to the skyline algorithm, the notes with smaller pitch value can be removed and a MIDI event sequence can be obtained. The pitch stored in the MIDI file is expressed in hexadecimal. According to the MIDI note coding table, it is converted to a decimal system, and each value corresponds to the corresponding note. The pitch of a note is represented by the semitone value; the semitone and pitch frequency have the corresponding relationship expressed by the following formula:

(7) $$\begin{array}{c} \text{Pitch}=69+12*\;\log_{2}\frac{f0}{144}, \end{array}$$

where 69 is the corresponding halftone value of international standard *A*, *f*0 represents the pitch frequency, and 144 is the frequency difference between two semitones.

#### 3.2.3. Pitch Feature Matching

**Figure 5:**
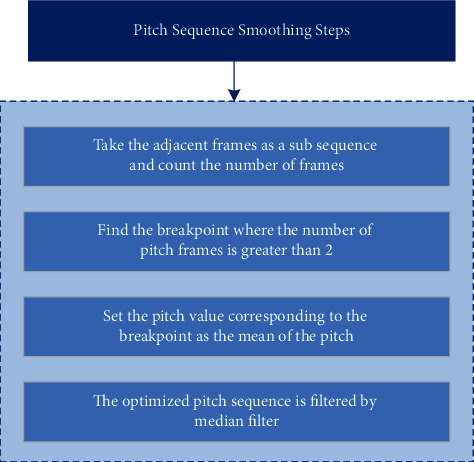
Steps of pitch sequence smoothing.

Assuming that each singer's goal is to sing the score accurately, the breaks should not last long and should be followed by a steady sub-sequence of pitches. In the pitch extraction, the audio signal is framed, and the pitch sequence obtained is also frame based. The breakpoint usually appears in the middle of two stationary signals, which only lasted 1–2 frames. Therefore, as shown in Figure 5, we can smooth the pitch sequence as follows:

1. The adjacent frames with equal pitch values in pitch sequence are regarded as a subsequence, and the number of frames is counted
2. After traversing the frame number of each pitch sub-sequence, the break point can be found where the frame number is between 1 and 2, and the pitch frame number before and after it is greater than 2
3. The pitch value corresponding to the breakpoint is set as the average pitch of the subsequences before and after the breakpoint
4. Median filter is used to optimize the pitch sequence

Assuming that the pitch sequences of the matching and template are respectively *X* and *Y*, where *p* represents pitch, *t* represents the number of frames, *k* is the number of subsequences with a continuous equal pitch in the sequence to be matched, and *v* is the number of notes in the template.

(8) $$\begin{array}{c} X\left(\left(p1,t1\right),\left(p2,t2\right),\left(p3,t3\right),\cdots ,\left(pk,tk\right)\right),\\ Y\left(\left(p1,t1\right),\left(p2,t2\right),\left(p3,t3\right),\cdots ,\left(pv,tv\right)\right). \end{array}$$

Replace frame matching with subsequence matching, then the calculation formula of distance is

(9) $$\begin{array}{c} \text{dist}\left(x_{i},y_{j}\right)=\sqrt{{\left(p_{i}\cdot t_{i}-p_{j}\cdot t_{j}\right)}^{2}}. \end{array}$$

The formula of pitch path matching is as follows:

(10) $$\begin{array}{c} w\left(x_{i},y_{j}\right)=m\left(\begin{array}{c} w\left(x_{i-1},y_{j}\right)+\text{dist}\left(x_{i-1},y_{j}\right)\\ w\left(x_{i-1},y_{j-1}\right)+\text{dist}\left(x_{i-1},y_{j-1}\right) \end{array}\right). \end{array}$$

## 4. Design of Music Tutorial System Based on Pitch Feature Matching

### 4.1. Input of Musical Score

This research will be applied as a learning tool, to achieve the function by expanding the components, the following part mainly introduces the spectrum component that is difficult to realize. Considering that only the notes appearing in the music score need to be input in solfeggio practice, in order to reduce the difficulty of input and improve the fluency, there are two input methods in this paper.

#### 4.1.1. Selective Input

Selective input means that the notes contained in the music score are provided in the input interface. After the user selects the input position, the system pops up the note selection interface, and the corresponding notes can be written into the staff and displayed.

#### 4.1.2. Input by MIDI Symbol

In the MIDI standard, the pitch and duration of notes are respectively expressed by numerical values, in which the pitch value corresponds to the keys of a piano, and the pitch within each 8 degree is, respectively, expressed by 12 numerical values according to the 12-tone law. Because the range of voices that people can sing is limited, only 0–8 sound zones are provided, and the duration of notes is set by the time slider.

### 4.2. Overall Structure of the System

**Figure 6:**
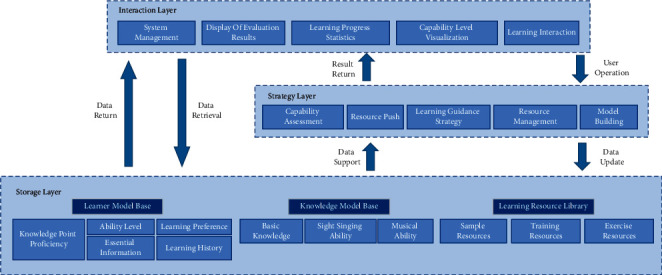
Overall structure of music tutorial system.

The music learning tutoring system includes a storage layer, strategy layer, and interaction layer. The storage layer is responsible for providing the databases needed by the system operation, including learner model base, knowledge model base, and learning resource base. The strategy layer is used for systematic decision-making and data analysis, it carries out personalized services such as learning resource push, resource management, intelligent learning guidance, learning effect statistics, and ability evaluation, with the data support of the storage layer. And, then it analyzes students' learning process in real time, so as to dynamically construct and update their learning models; the interaction layer is the man-machine interface of the system, including system management, evaluation results display, learning progress display, ability level visualization, and interaction of learning process. The overall structure of the system is shown in Figure 6.

The interaction layer only directly accesses the storage layer to obtain data during data retrieval, and other interactions need to analyze the requirements through the strategy layer and then return the results to the interaction layer after making a decision.

#### 4.2.1. Storage Layer

Learner model base: learner model base stores data of the learner model, including basic information of learners, solfeggio ability level, knowledge mastery, and learning history. Which provides the decision-making layer with the raw data needed for learning and analysis.

Knowledge model base: the knowledge model base is based on the knowledge model of solfeggio, including the main basic knowledge entities, solfeggio knowledge entities, and music knowledge entities.

Learning Resource Library: the resource library stores the examples, practice scores, and training scores needed in the learning process and establishes an index table according to the structure defined in the resource model, which facilitates the sorting and retrieval of resources.

#### 4.2.2. Strategy Level

Ability evaluation: ability evaluation consists of two parts: evaluation strategy and evaluation algorithm. The evaluation strategy is the solfeggio scoring index and ability level calculation method, and the evaluation algorithm is a solfeggio scoring algorithm based on pitch characteristics proposed in Chapter 3. This module is responsible for evaluating learners' performance in exercises and tests, then dynamically update the data of the submodel of ability level and knowledge mastery in the learner model.

Learning tutor strategy: learning tutor strategy includes three main functions: learning suggestions of knowledge points, skills in practice and training, and learning path adjustment. The learning suggestions of knowledge points come from the attribute fields in the knowledge model; skill information is used to make the analysis of the difficulties and error-prone points contained in the current learning resources and then gives learning suggestions in combination with specific teaching methods; learning path adjustment refers to the degree of difficulty of adjusting learning resources after dynamically analyzing learners' learning effects. For example, when learners learn a certain knowledge well for many times, the system will adjust the difficulty coefficient of the next learning resource recommendation.

Resource management: resource management consists of two main functions: resource import and resource sorting. Resource import is to provide learners with the function of customizing external resource import and online resource download; resource collation is to analyze the knowledge points and extract the difficulty features of the own resources or the resources imported by users, and it is also responsible for checking the validity of resource files.

Model construction: model construction mainly includes the learner model, the rules of knowledge point model construction, and the rules editing interface. Among them, rule editing is an interface to provide modification for the optimization of algorithms and models in the later system when there are enough user data.

#### 4.2.3. Interaction Layer

System management: system management is the common functional interface of the system, including user registration, login, information modification, system settings, and other functions.

Data visualization: data visualization is mainly the display and comparative analysis of data such as evaluation results, ability level, and learning progress, which consists of visualization components and data control. Visualization components are mainly various commonly used chart controls, such as graphs, radar charts, and data tables. Data control includes data request, data structure transformation, data update, report generation, and printing and is mainly responsible for data generation and output to be displayed.

### 4.3. Design of Interaction between Teachers and Students

**Figure 7:**
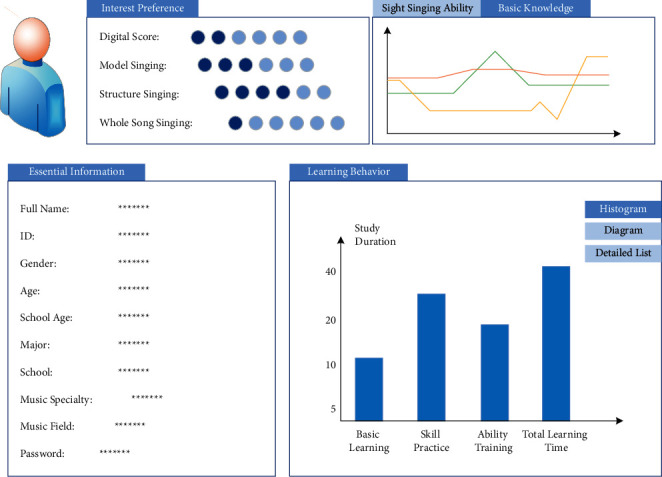
Learner interface.

The learner model is the data entity of learners in the intelligent tutoring system, which records all kinds of information related to learners and stores them in the database. In order to let learners know their learning status in real time, the system needs to provide a visual user interface to present the learner model, as shown in Figure 7.

There are two kinds of music score files in this system: pictures and MIDI, so there are two kinds of music score display modes in each learning resource, which can be switched freely. The title bar of the component displays music score information such as range, bar number, interval number, note number, chord number, and the playing button of music score audio. To meet the learning preferences of different learners, the system provides a variety of learning modes, some of which need the assistance of special tools, such as music score input tools in dictation and musical note playing tools in singing.

## 5. Conclusion

The recognition and distinction of music difficulty is the key to realize the individualization of music teaching resources. Only by accurately recommending teaching resources with difficulty and the ability for learners, can they make effective use of resources, quickly master the knowledge and avoid the loss of learning interest. Therefore, this paper summarizes and analyzes the problem of pitch feature extraction in music teaching. By combining with the characteristics of music pitch, the features of high-pitched content in music teaching resources are matched based on deep learning. Then, the algorithm of pitch feature extraction in MIDI music score files is put forward, and the pitch feature matching model is constructed. Finally, a music tutoring system based on pitch feature matching is designed, including the design of music score learning tools, the overall structure of the system, and the interaction between teachers and students, which is helpful to realize the individualized teaching of music solfeggio, and further improve the music teaching model and realize intelligent and personalized music learning.

## Data Availability

The dataset can be accessed from the author upon request.
